# Long-Term Effects of Liming on Health and Growth of a Masson Pine Stand Damaged by Soil Acidification in Chongqing, China

**DOI:** 10.1371/journal.pone.0094230

**Published:** 2014-04-11

**Authors:** Zhiyong Li, Yanhui Wang, Yuan Liu, Hao Guo, Tao Li, Zhen-Hua Li, Guoan Shi

**Affiliations:** 1 College of Agriculture, Henan University of Science and Technology, Luoyang, China; 2 Institute of Forest Ecology, Environment and Protection, Chinese Academy of Forestry, Beijing, China; 3 University of Chinese Academy of Sciences, Beijing, China; 4 Institute of Desertification Studies, Chinese Academy of Forestry, Beijing, China; 5 Hebei University, Baoding, China; Institute for Plant Protection (IPP), CNR, Italy

## Abstract

In the last decades, the Masson pine (*Pinus massoniana*) forests in Chongqing, southwest China, have increasingly declined. Soil acidification was believed to be an important cause. Liming is widely used as a measure to alleviate soil acidification and its damage to trees, but little is known about long-term effects of liming on the health and growth of declining Masson pine forests. Soil chemical properties, health condition (defoliation and discoloration), and growth were evaluated following application of limestone powder (0 (unlimed control), 1, 2, 3, and 4 t ha^−1^) in an acidified and declining Masson pine stand at Tieshanping (TSP) of Chongqing. Eight years after liming, in the 0–20 cm and 20–40 cm mineral soil layers, soil pH values, exchangeable calcium (Ca) contents, and Ca/Al molar ratios increased, but exchangeable aluminum (Al) levels decreased, and as a result, length densities of living fine roots of Masson pine increased, with increasing dose. Mean crown defoliation of Masson pines (dominant, codominant and subdominant pines, according to Kraft classes 1–3) decreased with increasing dose, and it linearly decreased with length densities of living fine roots. However, Masson pines (Kraft classes 1–3) in all treatments showed no symptoms of discoloration. Mean current-year twig length, twig dry weight, needle number per twig, needle length per twig, and needle dry weight per twig increased with increasing dose. Over 8 years, mean height increment of Masson pines (Kraft classes 1–3) increased from 5.5 m in the control to 5.8, 6.9, 8.3, and 9.5 m in the 1, 2, 3, and 4 t ha^−1^ lime treatments, and their mean DBH (diameter at breast height) increment increased from 3.1 to 3.2, 3.8, 4.9, and 6.2 cm, respectively. The values of all aboveground growth parameters linearly increased with length densities of living fine roots. Our results show that liming improved tree health and growth, and these effects increased with increasing dose.

## Introduction

Atmospheric emissions of acidifying compounds resulting from anthropogenic activities since the beginning of the industrial revolution have led to extensive soil acidification in terrestrial ecosystems [1]–[3]. Soil acidification has been blamed for causing indirect forest damage in Europe, North America, and Asia in the past few decades [4]–[10]. Some experiments have been implemented with liming in forests in Europe and the northeastern United States. Investigations on forest liming have shown that it enhanced cation exchange capacity (CEC), base saturation (BS), and contents of exchangeable Ca and Mg in the forest floor and the mineral soil, while Al levels and acidity decreased [11]–[15]. Effects of liming on the growth of tree fine roots were reported to be positive [16]–[18], although some researchers almost found the contrary, depending on tree species, tree age, site, soil conditions, form and dose of liming, and the time lapse since liming [19]–[21]. In contrast to expectations, however, liming has not made an overall improvement in aboveground growth of trees; instead, it has often produced either no effect or a detrimental effect on stand growth [22]–[26]. But, positive effects of liming on stand growth have also been observed [27]–[29]. The effect of liming on tree growth is dependent on the soil chemical properties, particularly the deficiency of Ca and Mg [29]. In addition, the reaction of forest stands to liming can be affected by such factors as tree species and age [30].

Masson pine (*Pinus massoniana*) is one of the most widespread forest species across subtropical China and it is highly sensitive to acid deposition [31]. Chongqing, located in the upper reaches of the Yangtze River, is an industrial and commercial municipality in southwest China. Total areas of pure Masson pine forests are in excess of 1.2 million hectares and estimated to account for more than 50% of the total forests in the region [32]. Obviously, this conifer species plays a very important role in protecting the environment of the middle and lower reaches of the Yangtze River. On the other hand, Chongqing ranks amongst the regions with the highest levels of acid deposition in China [33]–[35]. Due to extensive use of coal with high sulphur (S) contents, basin topography conducive to the accumulation of air pollutants, and low wind velocity, Chongqing has undergone severe deposition of acidifying S compounds since the late 1970s, when the Chinese Economic Reform Policy started [1], [33], [36]–[38]. More than half of 40 districts and counties within the region was designated as being part of so-called acid rain control areas, i.e. parts of China where pH value of rain water is lower than 4.5 and sulfur deposition exceeds the critical load, and the acid rain problem has been addressed by the national authorities [39]. The soil of Masson pine forests in Chongqing is generally acidic, with low amounts of exchangeable Ca and high amounts of exchangeable Al [33], [35], [40], [41]. Masson pine is highly sensitive to Al [42]. Since the 1980s, large patches of Masson pine forest in some areas have exhibited serious decline symptoms, mainly including fine root death, tip necrosis of needles, thinned crown, reduced needle length, premature needle abscission, dieback of twigs and branches, and reduced growth, which was believed to be caused by soil acidification [4], [33], [35], [43], [44].

A common measure to counteract soil acidification is liming of soils [45]–[47]. In the last decades, however, only a few studies have been conducted to investigate the effects of liming on Masson pine forests growing on acidified soil, mainly with a single lime dose over relatively short time spans. For example, Du and Tian [44] found increased pH values in the 0–25 cm mineral soil on a limed young Masson pine forest 2 years after liming. Huang et al. [48] observed increased Ca contents in current-year needles at limed plots established in a 40-year-old pure Masson pine forest about 1 year after liming with 3 t ha^−1^ limestone powder. Up to now, little is known about the long-term effects of different lime doses on Masson pine. Thus, fifteen permanent plots treated with five doses of limestone powder were set up in a Masson pine stand damaged by soil acidification in Chongqing in 2004, and an investigation was carried out 8 years after liming. The aim of this work was to assess the effects of the five doses on the health and growth of Masson pine.

## Study Sites and Methods

### Site description

This study was conducted at Tieshanping (TSP) (29°38′N, 106°41′E, 512–579 m a.s.l.). TSP, covering about 1200 ha, is an almost pure Masson pine forest located on a sandstone ridge approximately 25 km northeast from the urban centre of Chongqing (28°10′–32°13′N, 105°17′–110°11′E) in southeast Sichuan Basin, southwest China. Masson pines have been planted since the early 1960s. The study site has a subtropical humid climate, with a mean annual air temperature of 18°C, a mean annual precipitation of 1100 mm that mainly occurs from April to October, and a mean annual relative humidity of 80%. TSP has been experiencing a long-term severe acid deposition, with low mean annual pH (4.0–4.2) and high frequency of acid rain (rainfall of pH<5.6 being about 90.0%) [35], [38], [49]. High deposition of S has been frequently reported. For example, during 2002 and 2003, throughfall S input was reported to be 16.0 g S m^−2^ a^−1^, and throughfall N input was measured at 4.0 g N m^−2^ a^−1^ [50]. The forest soil is yellow mountain soil (Haplic Acrisol, WRB) developed on sandstone and prone to acidification. The mean mineral soil depth is between 40 and 80 cm, and the soil was rather homogeneous [33], [34]. This acidic soil is representative for Chongqing and characterized by low levels of exchangeable Ca and high levels of exchangeable Al [33], [35], [41]. In the last decades the Masson pine forest ecosystem has declined due to severe soil acidification [33], [35].

### Plot establishment and liming treatments

In early May 2004, we selected a 26-year-old Masson pine stand, damaged (defoliation >25%) by soil acidification [35] for this limestone powder dose experiment. This stand grew on almost level ground and was composed of evenly-spaced and similarly-sized Masson pines, which were similar in defoliation. Therein, only a few scattered individuals of undergrowth species, e.g. *Schima superba*, *Cunninghamia lanceolata*, *Schoepfia jasminodora*, and *Lithocarpus glaber* grew. The density of Masson pines for this stand was 1192 trees ha^−1^. In early June 2004, the mean defoliation, discoloration, height, and DBH of Masson pines (dominant, codominant and subdominant pines, Kraft classes 1–3) for this stand were 34%, 1%, 13.3 m, and 15.5 cm, respectively.

The trial was established in this stand as a randomized complete block experiment with five limestone powder (49.5% CaO, particle size <0.25 mm) treatments (0 (unlimed control), 1, 2, 3, and 4 t ha^−1^) and three replicates. There were total fifteen plots. Each plot measured 10 m×10 m, with about 1 m buffer strips around each plot. The overall size of the stand within which all plots were installed was about 0.3 ha. Limestone powder was broadcast one time and evenly on the forest floor by hand in early June 2004.

### Soil and living fine root sampling and measurements

In mid June 2012, two dominant Masson pines (Kraft class 1) were selected near the centre of each plot for sampling. Humus and soil were collected from the east, south, west, and north directions 1.0 m away from the main stem of each selected tree. Samples of the humus layer and the 0–20 cm, 20–40 cm and 40–60 cm mineral soil layers were taken using a cylindrical auger with an inner diameter of 10 cm and a height of 25 cm. The four samples collected around each selected tree in each plot were pooled to produce one composite sample per layer. In all, there were 120 composite samples representing five treatments and three field replicates. All composite samples were placed in plastic bags and transported to the laboratory, where they were bulked and crumbled manually. Living roots of different diameters of Masson pine were picked out according to the characteristics of appearance, color, elasticity, and odor [35] and placed into 0.2-mm sieves using tweezers. Meanwhile, roots of other plant species and gravel were removed. The living roots of Masson pine were carefully cleaned and washed in slow running water to remove soil from roots, spread out on clean sheets of paper for 5–10 minutes in the shade, kept in sealed bags, and stored at −20°C until later measurements. After being air-dried at room temperature (25°C), all soil samples were ground, passed through a 2-mm sieve, and stored at 4°C in the dark before chemical analysis.

Soil pH was determined in a 1∶2.5 slurry of soil∶1 M KCl solution using a combination glass electrode, after shaking for 0.5 h and then equilibrating for 0.5 h. The accuracy was 0.01 pH. Exchangeable Ca and Al were extracted with 1 M KCl solution and determined using an atomic absorption spectrometer (AAnalyst 300, Perkin-Elmer, CT, USA). The Ca/Al molar ratios were calculated from exchangeable Ca and Al contents of all humus and soil samples. Lengths of living fine roots (diameter ≤2 mm) were measured using an automated root scanning and analysis system (WinRHIZO, Regent Instruments Inc., Canada). Length densities (m m^−3^) of living fine roots were calculated from all samples according to the volumes of the humus and soil samples. During sampling and measurements, polypropylene gloves were used to avoid contamination. In this study, we chose to analyze the soil chemical properties and fine root growth of Masson pine in the 0–20 cm and 20–40 cm mineral soil layers where the roots were mainly distributed [33], [35].

### Health and growth monitoring

In early June 2004 and in early June 2012, the assessments of crown health condition (defoliation and discoloration), height, and DBH were performed twice on all dominant, codominant and subdominant Masson pines (Kraft classes 1–3) per plot according to the ICP–Forests manual [51]. Defoliation and discoloration in the assessable crown were estimated based on 5% classes as compared with a local reference tree, which is defined as the healthiest tree that could grow at a particular site, taking into account factors such as altitude, latitude, tree age, site conditions and social status. The local reference tree has 0% defoliation and 0% discoloration. Pines with defoliation of 0–10%, >10–25%, >25–60%, >60–<100%, and 100% are classified as no defoliation, slight defoliation, moderate defoliation, severe defoliation, and death, respectively. Pine trees with defoliation >25% were classified as “damaged” [52]. Pine trees with discoloration of 0–10%, >10–25%, >25–60%, and >60% are categorized as no discoloration, slight discoloration, moderate discoloration, and severe discoloration, respectively. Tree height was measured with a Haga hypsometer, and DBH using a diameter tape.

At TSP, current-year needles make up the majority of tree foliage. In early June 2012, five current-year twigs with needles were sampled from the upper third of the crown on the south side of each of the two selected trees near the centre of each plot. The five current-year twigs with needles collected from each selected tree per plot were pooled to form a sample. Thirty samples were transported in clean plastic bags to the laboratory, where for each sample we separated needles from twigs, and measured twig length, twig dry weight, needle number per twig, needle length per twig, and needle dry weight per twig. Twig and needle lengths were measured to the nearest millimeter. The samples were ovendried at 60°C for about one week to a constant weight and weighed. Mean twig lengths, mean twig dry weights, mean needle numbers per twig, mean needle lengths per twig, and mean needle dry weights per twig were calculated from all samples.

### Statistical analysis

The data were expressed as mean ± standard deviation (SD). They were assessed using one-way analysis of variance (ANOVA) with the SPSS 12.0 software package. The fixed model was used in ANOVA. Multiple comparisons of means among treatments were performed using Duncan test. *P*<0.05 was considered significant.

## Results

### Soil chemistry

**Figure 1:**
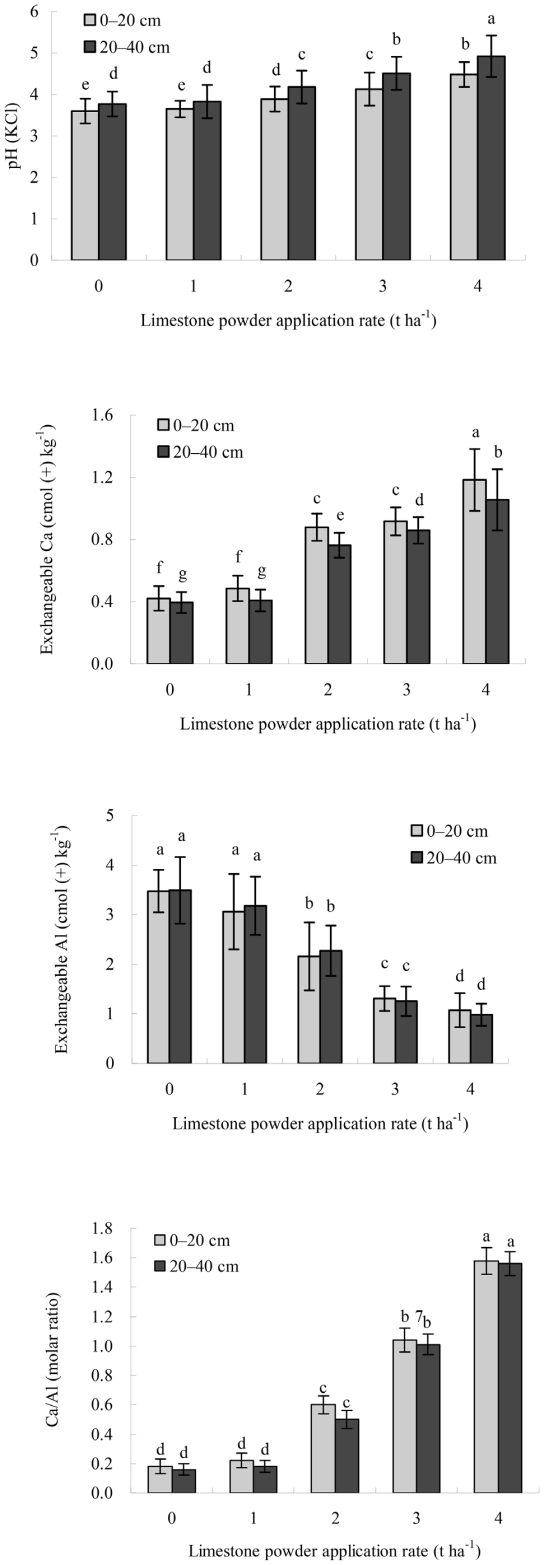
Soil pH values, exchangeable Ca contents, exchangeable Al levels, and Ca/Al molar ratios in the 0–20 cm and 20–40 cm of the Masson pine stand 8 years after liming. Error bars represent the standard deviation (SD) of the mean. Different letters above the error bars indicate significant differences at the 0.05 level (ANOVA and Duncan's multiple range test), n = 3.

Liming increased the pH, exchangeable Ca, and Ca/Al molar ratio, but decreased the exchangeable Al in the 0–20 cm and 20–40 cm mineral soil layers (Fig. 1). The pH, exchangeable Ca, and Ca/Al molar ratio in the same layers were found to be in the following order of lime treatments: 4 t ha^−1^>3 t ha^−1^>2 t ha^−1^>1 t ha^−1^>0 t ha^−1^, while the exchangeable Al exhibited the reverse pattern. Overall, there were no statistical differences in the soil chemical parameters in the same layers between the 0 and 1 t ha^−1^ lime treatments, whereas significant differences were observed among the 1, 2, 3, and 4 t ha^−1^ lime treatments (*P*<0.05).

### Fine root growth

**Figure 2:**
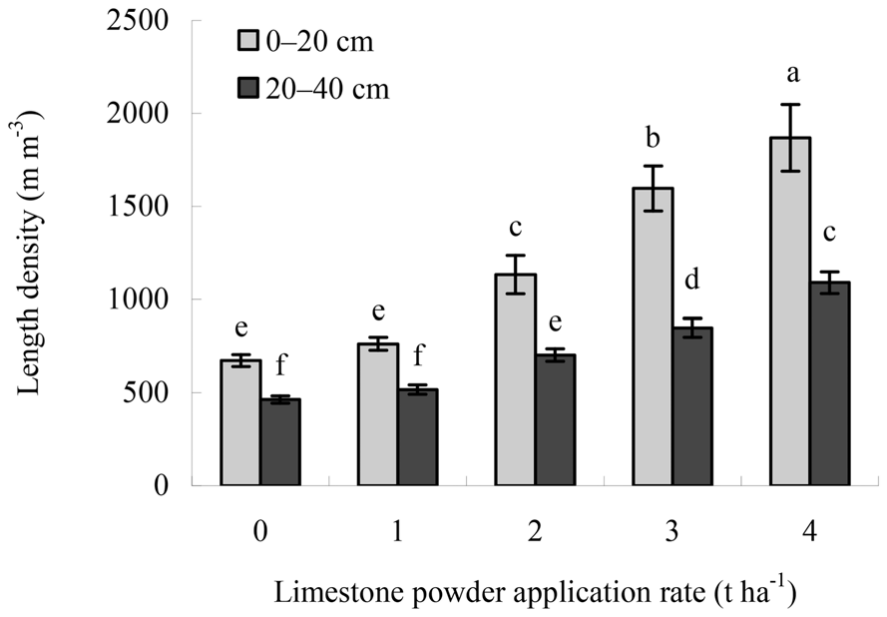
Length densities of living fine roots of Masson pine in the 0–20 cm and 20–40 cm of the Masson pine stand 8 years after liming. Error bars represent the SD of the mean. Different letters above the error bars indicate significant differences at the 0.05 level (ANOVA and Duncan's multiple range test), n = 3.

Liming improved the growth of fine roots of Masson pine in the 0–20 cm and 20–40 cm mineral soil layers (Fig. 2). The length densities of living fine roots in the same layers were observed to be in the following order of lime treatments: 4 t ha^−1^>3 t ha^−1^>2 t ha^−1^>1 t ha^−1^>0 t ha^−1^. There were no statistical differences in the length density in the same layers between the 0 and 1 t ha^−1^ lime treatments, whereas significant differences were found among the 1, 2, 3, and 4 t ha^−1^ lime treatments (*P*<0.05). The length densities of living fine roots were significantly positively correlated with the pH values, exchangeable Ca contents, and Ca/Al molar ratios, but significantly negatively with the exchangeable Al levels in both soil layers (Table 1).

**Table 1:** Relationships between the length densities (*y*, m m^−3^) of living fine roots and the soil chemical properties (*x*) in the 0–20 cm and 20–40 cm.

Soil horizon (cm)	*x*	Unit of *x*	Regression equation *y* = a*x*+b	Correlation coefficient *R* ^2^	Degree of freedom *df*	*F*-value	*P*-value
0–20	pH (KCl)	–	*y* = 1408.5*x*-4356.6	0.9741	4	150.44	<0.05
	Exchangeable Ca	cmol (+) kg^−1^	*y* = 1562.4*x*-7.207	0.9212	4	46.76	<0.05
	Exchangeable Al	cmol (+) kg^−1^	*y* = −485.98*x*+2284.2	0.9707	4	132.52	<0.05
	Ca/Al (molar ratio)	–	*y* = 866.51*x*+579.7	0.9757	4	160.61	<0.05
20–40	pH (KCl)	–	*y* = 528.91*x*-1519.6	0.9970	4	1329.33	<0.05
	Exchangeable Ca	cmol (+) kg^−1^	*y* = 864.05*x*+122.69	0.9564	4	87.74	<0.05
	Exchangeable Al	cmol (+) kg^−1^	*y* = −220.62*x*+1217.6	0.9342	4	56.79	<0.05
	Ca/Al (molar ratio)	–	*y* = 86.627*x*+456.97	0.9913	4	455.77	<0.05

### Crown condition

**Figure 3:**
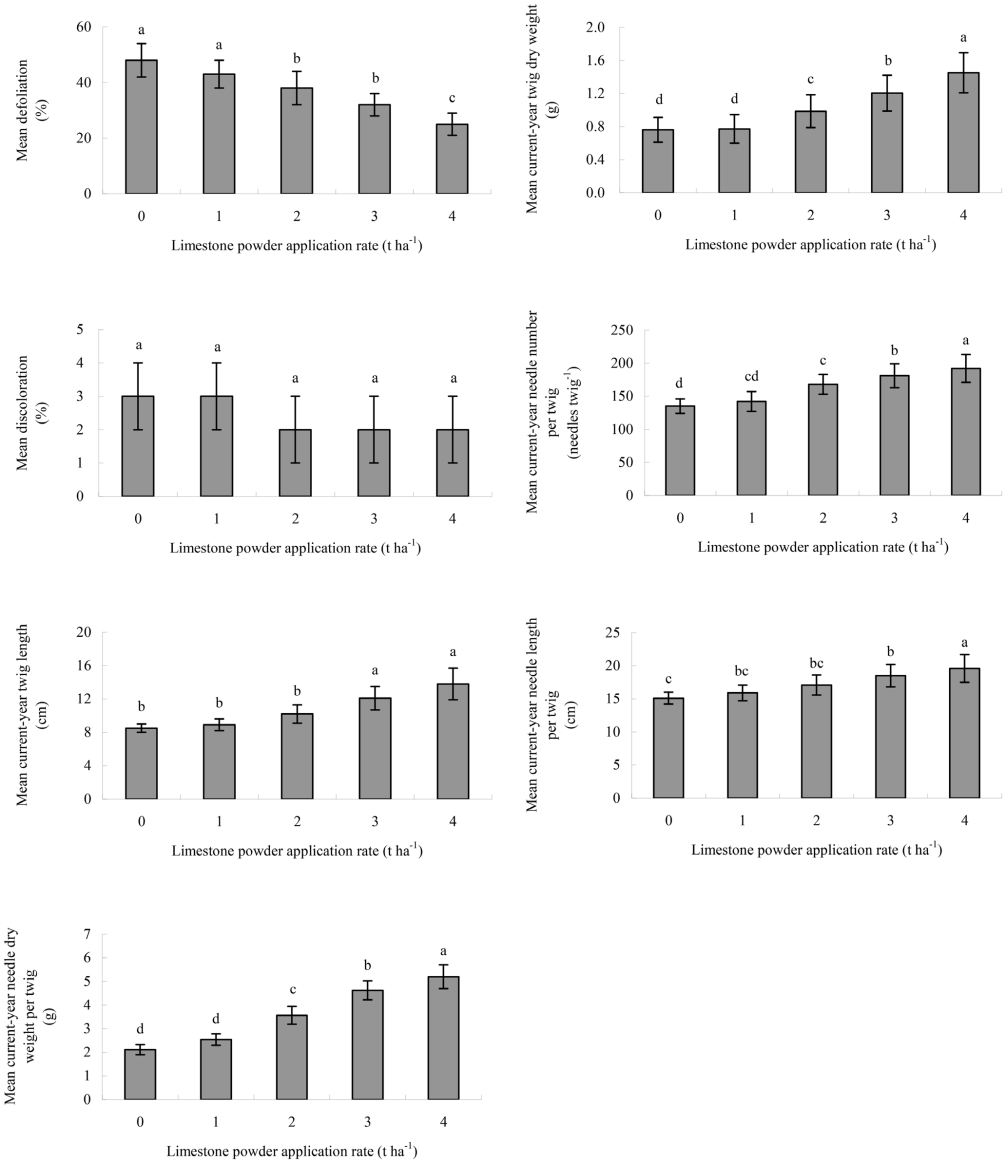
Crown condition of pines (Kraft classes 1–3) in the Masson pine stand 8 years after liming. Error bars represent the SD of the mean. Different letters above the error bars indicate significant differences at the 0.05 level (ANOVA and Duncan's multiple range test), n = 3.

Liming decreased the defoliation of Masson pines (Kraft classes 1–3), but increased their current-year twig and needle growth (Fig. 3). The mean defoliation varied with lime treatments, following an order of 0 t ha^−1^>1 t ha^−1^>2 t ha^−1^>3 t ha^−1^>4 t ha^−1^. In contrast, the mean current-year twig length, twig dry weight, needle number per twig, needle length per twig, and needle dry weight per twig showed the reverse behavior. The mean defoliation of Masson pines (Kraft classes 1–3) in the 4 t ha^−1^ lime treatment was 25%, which is the lower limit of the moderate defoliation range (>25%–60%). However, the mean discoloration of Masson pines (Kraft classes 1–3) in all treatments lay within no discoloration range (0–10%). The mean defoliation was significantly negatively correlated with the length densities of living fine roots in both soil layers, but the values of current-year twig and needle growth parameters were significantly positively correlated with them (Table 2).

**Table 2:** Relationships between the crown condition (*y*) and the length densities (*x*, m m^−3^) of living fine roots in the 0–20 cm and 20–40 cm.

Soil horizon (cm)	*y*	Unit of *y*	Regression equation *y* = a*x*+b	Correlation coefficient *R* ^2^	Degree of freedom *df*	*F*-value	*P*-value
0–20	Mean defoliation	%	*y* = −0.0172*x*+57.915	0.9720	4	138.86	<0.05
	Mean current-year twig length	cm	*y* = 0.0043*x*+5.5405	0.9903	4	408.37	<0.05
	Mean current-year twig dry weight	g	*y* = 0.0006*x*+0.354	0.9845	4	254.06	<0.05
	Mean current-year needle number per twig	Needles twig^−1^	*y* = 0.0465*x*+107.49	0.9658	4	112.96	<0.05
	Mean current-year needle length per twig	cm	*y* = 0.0035*x*+12.984	0.9892	4	366.37	<0.05
	Mean current-year needle dry weight per twig	g	*y* = 0.0025*x*+0.5518	0.9922	4	508.82	<0.05
20–40	Mean defoliation	%	*y* = −0.0351*x*+62.617	0.9816	4	213.39	<0.05
	Mean current-year twig length	cm	*y* = 0.0087*x*+4.4043	0.9890	4	359.64	<0.05
	Mean current-year twig dry weight	g	*y* = 0.0012*x*+0.2003	0.9920	4	496.00	<0.05
	Mean current-year needle number per twig	Needles twig^−1^	*y* = 0.0936*x*+95.809	0.9456	4	69.53	<0.05
	Mean current-year needle length per twig	cm	*y* = 0.0071*x*+12.075	0.9776	4	174.57	<0.05
	Mean current-year needle dry weight per twig	g	*y* = 0.0051*x*-0.068	0.9631	4	104.40	<0.05

### Height and DBH growth

Liming increased the height and DBH of Masson pines (Kraft classes 1–3) from early June 2004 to early June 2012. Among all treatments, the 4 t ha^−1^ lime treatment was the greatest in terms of both the mean height increment and the mean DBH increment of Masson pines (Kraft classes 1–3), it was followed by, in decreasing order, the 3, 2, 1, and 0 t ha^−1^ lime treatments. The mean height increments of Masson pines (Kraft classes 1–3) were 5.5±0.4, 5.8±0.5, 6.9±0.5, 8.3±0.6, and 9.5±0.6 m in the 0, 1, 2, 3, and 4 t ha^−1^ lime treatments, and their mean DBH increments 3.1±0.2, 3.2±0.2, 3.8±0.3, 4.9±0.4, and 6.2±0.4 cm, respectively. There were no statistical differences in both parameters between the 0 and 1 t ha^−1^ lime treatments, but significant differences were detected among the 1, 2, 3, and 4 t ha^−1^ lime treatments (*P*<0.05). The mean height increment and mean DBH increment were significantly positively correlated with the length densities of living fine roots in the 0–20 cm and 20–40 cm mineral soil layers (Table 3).

**Table 3:** Relationships between the mean height and DBH increments (*y*) and the length densities (*x*, m m^−3^) of living fine roots in the 0–20 cm and 20–40 cm.

Soil horizon (cm)	*y*	Unit of *y*	Regression equation *y* = a*x*+b	Correlation coefficient *R* ^2^	Degree of freedom *df*	*F*-value	*P*-value
0–20	Mean height increment	m	*y* = 306.23*x*-998.03	0.9951	4	812.33	<0.05
	Mean DBH increment	cm	*y* = 387.93*x*-438.03	0.9565	4	87.95	<0.05
20–40	Mean height increment	m	*y* = 150.05*x*-356.26	0.9893	4	369.83	<0.05
	Mean DBH increment	cm	*y* = 192.68*x*-92.876	0.9771	4	170.67	<0.05

## Discussion

Liming in early June 2004 increased the soil pH and exchangeable Ca and decreased the exchangeable Al to a 40 cm depth on the Masson pine stand; this effect increased with increasing dose. It is worth noting that in 0–20 cm and 20–40 cm in the 4 t ha^−1^ lime treatment the soil pH values increased to 4.48 and 4.92, respectively (Fig. 1), which are very near to or in the optimum pH (KCl) range (4.50–6.00) for the growth of Masson pine [53]. As a result, amelioration of the decline of the Masson pine stand has been observed 8 years after liming; this effect also increased with increasing dose. From the viewpoint of forest health restoration, the dose of 4 t ha^−1^ limestone powder is suggested, since this dose induced the best health and growth of Masson pine. More long-term observations are needed to fully assess the effects of liming on the health and growth of the Masson pine stand.

Fine roots as uptake organs to sustain aboveground health and growth of forests are susceptible to changes in the soil environment [54]. Due to soil acidification, toxic Al is released, which adversely impacts the growth of fine roots and inhibits the uptake of water and cations [55]–[57]. Al stress on the growth of fine roots is governed by the Ca/Al molar ratio [58]. According to Cronan and Grigal [56], the Ca/Al molar ratio in soil solution provided an indicator for identification of thresholds beyond which the risk of fine root damage from Al stress and nutrient imbalances increased. They estimated that there was a 50% risk of Al toxicity when the Ca/Al molar ratio was as low as 1.0, a 75% risk when the ratio was as low as 0.5, and nearly a 100% risk when the ratio was as low as 0.2.

This study showed that in 0–20 cm and 20–40 cm the Ca/Al molar ratios increased with increasing dose (Fig. 1) and the length densities of fine roots of Masson pine linearly increased with the ratios (Table 1). Al stress on the growth of fine roots of Masson pine was probably excluded in both layers in the 3 and 4 t ha^−1^ lime treatments, where the Ca/Al molar ratios were >1.0, and the best growth of fine roots was found in the 4 t ha^−1^ lime treatment. However, risks seemed to exist in both layers in the 0, 1, and 2 t ha^−1^ lime treatments, where the Ca/Al molar ratios were <1.0. The stress indication was very noticeable in both layers in the 0 and 1 t ha^−1^ lime treatments, where the Ca/Al molar ratios were ≤0.22, and the worst growth of fine roots was observed in the 0 t ha^−1^ lime treatment (Figs. 1 and 2). The application of limestone powder ameliorated Al toxicity on fine roots of Masson pine; this effect increased with increasing dose.

Liming ameliorates upper soil acidity in a relatively short term, but is generally slow in reducing deeper soil acidity [29], [47]. The movement of lime to greater depths depends on site, soil type, form of lime, timing and rate of liming, and weather conditions [20], [59]–[62]. Matzner et al. [63] showed a clear increase in exchangeable Ca and Mg in the 0–50 cm mineral soil depth in an experiment in *Fagus sylvatica* and *Picea abies* stands about 10 years after fertilization of NH_4_NO_3_ and KCl followed by Ca and Ma fertilization by 2 liming applications. Moore et al. [64] reported that in an uneven-aged *Acer saccharum* forest 10 years after liming, exchangeable Ca and Mg contents down to 20 cm depth increased obviously with the amount of added lime (powdered and pelletized dolomitic lime) while Ca contents in 20–40 cm depth were unaffected. Guckland et al. [47] found a significant increase in exchangeable Mg in the forest floor and exchangeable Ca in the 0–20 cm mineral soil depth in stands of *P. abies*, *F. sylvatica*, *Quercus petraea/Q. robur*, and *Pinus sylvestris* 2–9 years after one-time liming with 3–5 t ha^−1^ lime (mainly calcite and dolomite), and they suggested that the element input, CEC, C stock of the forest floor, and groundwater recharge were the most important parameters connected with the change of Ca and Mg stocks after liming in 0–10 cm, but in 10–20 and 20–40 cm, the C concentration of this depth and the year after first liming gained more influence. In our study conducted at TSP in the humid subtropical zone, in the 0–40 cm mineral soil depth of the limed Masson pine stand 8 years after one-time liming with 2–4 t ha^−1^ limestone powder, significant effects of liming were observed on the chemical properties and the growth of fine roots (Figs. 1 and 2). The fast downward movement of dissolved Ca cations into deeper mineral soil depth (the 20–40 cm) might be mainly related to the amount of finely ground limestone powder applied, rather thin forest floor layer (approximately 1-cm-thick humus layer), and relatively high annual and seasonal rainfall in Chongqing [33], [34]. Undoubtedly, the promotion of the growth of fine roots of Masson pine in the 20–40 cm in the 2–4 t ha^−1^ lime treatments, especially the application of high lime doses, is beneficial to enhancing tree resistance to drought and frost.

The growth of aboveground parts (stem, branches, twigs, and leaves) of plants is coordinated with the growth of belowground part (roots) [20], [65]–[67]. This lime-induced stimulation of the growth of fine roots of Masson pine favored Ca uptake by trees [48], resulting in the improvement of aboveground health and growth of trees (Fig. 3). The results in this study showed that the mean tree crown defoliation linearly decreased with the length densities of living fine roots in both soil layers, whereas the values of all aboveground growth parameters linearly increased with them (Tables 2 and 3). Pine trees with more fine roots grew more healthily and faster than those with less fine roots.

In summary, in the acidified and declining Masson pine stand of Chongqing, southwest China, application of limestone powder proved to be effective for ameliorating the soil acidity, improving the crown health condition, and stimulating the tree growth; these effects increased as the amount of applied limestone powder increased.
